# Correlates of hand grip strength in a cohort of older Nigerian Africans: Findings from the population-based VALIANT project

**DOI:** 10.1016/j.tjfa.2025.100068

**Published:** 2025-09-03

**Authors:** Rufus O. Akinyemi, Oladotun V. Olalusi, Gabriel O. Ogunde, Tolulope O. Akinyemi, Joseph O. Yaria, Olabode Oguntiloye, Ayotomiwa Fagbemi, Eniola O. Cadmus, Femi O. Popoola, Mayowa Ogunronbi, Dorcas Olujobi, Olaoluwa Famuyiwa, Joshua O. Akinyemi, Mayowa O. Owolabi, Roman Romero-Ortuno, Adesola Ogunniyi, Raj Kalaria, Brian Lawlor

**Affiliations:** a Neuroscience and Aging Research Unit, Institute of Advanced Medical Research and Training, College of Medicine, University of Ibadan, Ibadan, Nigeria; b Department of Neurology, University College Hospital, Ibadan, Oyo, Nigeria; c College of Medicine, University of Ibadan, Ibadan, Oyo, Nigeria; d Lead City University, Ibadan, Oyo, Nigeria; e Department of Epidemiology and Medical Statistics, College of Medicine, University of Ibadan, Ibadan, Nigeria; f Global Brain Health Institute, Trinity College, Dublin, Ireland; g African Dementia Consortium, Ibadan, Ibadan, Nigeria; h Translational and Clinical Research Institute, Newcastle University, Campus for Ageing & Vitality, Newcastle Upon Tyne NE4 5PL, United Kingdom; i Global Brain Health Institute, Trinity College Dublin, Dublin, Ireland; j Trinity College Institute of Neuroscience, School of Psychology, Trinity College Dublin, Dublin, Ireland; k St James Hospital, Dublin, Ireland

**Keywords:** Hand grip strength (HGS), Frailty, Nigerian-Africans, Indigenous Africans, Cognitive health, Physical health, Physical fitness, LMICs, Muscular strength

## Abstract

Previous evidence suggests there are racial, genetic, and geographic differences in hand grip strength (HGS) underlying the need to define population-specific normative values and their determinants. We determined the normative values of HGS and investigated its correlates in an urban community sample in Nigeria. We assessed 607 participants from the Vascular heAlth, fraiLty, and cognItion in Ageing Nigerians sTudy [VALIANT] – a population-based cohort of 1031 older persons in Ibadan, a city in Southwestern Nigeria. They were recruited through a multi-stage, stratified cluster random sampling method. HGS was defined as the highest recorded/maximum HGS (max. HGS) of both hands. The determinants (β coefficient, p values) of HGS were identified using linear regression model. The mean (SD) age of the participants was 64.6 (11.5) with 67.8 % females. The mean (SD) max. HGS (in kg) of the study population was 19.65±10.16 overall; 24.51(14.01) among males and 17.31(6.51) among females (*p* < 0.001). Overall, female sex (β=-6.38, *p* < 0.001), clinical frailty (β=-1.35, *p* < 0.001), and baseline MoCA scores (β=0.37, p 0.001) were independently associated with max. HGS. In the subgroup analysis, presence of hypertension (β=2.64, *p* < 0.001) and diabetes mellitus (β=-2.61, p 0.05) were independently associated with max. HGS among females, but not males. Our findings buttress the intricate link between overall physical, cardiometabolic as well as cognitive health in this unique West African population.

## 1 Background

Frailty is an important construct in geriatric medicine. It is believed that increased protein oxidation occurring in older age leads to cellular dysfunction [1] and reduction in tissue performance, subsequently causing a decline in function and frailty [2]. According to the Fried model, hand grip strength (HGS) is one of the five substrates of frailty [3]. While it is known that HGS reduces with age [4], varies according to diet, place of residence (geography) [5], body composition and co-morbid conditons [[6], [7], [8]], there is a growing need for representative data from low-resource settings [4]. In a recent study involving 2.4 million people from 69 countries, normative data on HGS were only available from two low-income countries [4]. Prior authors [6,9] have documented lower HGS among predominantly young-middle aged Nigerians compared to their Western counterparts [6]. These findings, while similar to observations from a population-based cohort of older adults in rural Ghana [7], were lower than findings from a study of older adults in Southern Africa [10], buttressing the wide variability in HGS across populations.

Just like other complex traits, HGS with well-documented phenotypic and genetic variation is influenced by multiple genetic variants of small effect sizes [[11], [12], [13], [14]]. Nigeria, being the most populous nation in Africa, is home to over 250 million people of diverse ethno-racial and ancestral backgrounds [15]. Characterization of HGS among the older Nigerian population may expand the frontiers of understanding the phenotypic variation of HGS among indigenous Africans. This is important as the Nigerian population is rapidly aging with an increasing burden of cardiovascular disease. Therefore, the need to have simple, cost-effective and practical biomarkers of frailty for use in a local population cannot be over-emphasized. This is necessary for the development of health and social care interventions and improved service delivery to maintain functional independence in older age. We determined the normative values of HGS and investigated its determinants in a semi-urban Nigerian population of older adults.

## 2 Methods

We analysed baseline data from the Vascular heAlth, CognItion in aging Nigerians sTudy [VALIANT] – a population-based cohort of older adults, enrolled from an urban settlement in Ibadan, a city in Southwestern, Nigeria. THE VALIANT Study is a longitudinal community-based cohort study aimed at exploring the association between cardiovascular health, cognition, and frailty in an indigenous Nigerian cohort. A multi-stage cluster sampling method was employed to recruit the study participants from Yemetu, an urban settlement in Ibadan North local government area (LGA). Using the African Rigorous Innovative Stroke Epidemiological Surveillance (ARISES) database [16] as a sampling frame, two wards (Ward 3 & 4) were selected. ARISES is an ongoing observational cohort study in selected wards in Ibadan North and Ibarapa Central local government areas in Oyo State, Nigeria. Wards 3 & 4 were purposively selected for the VALIANT study because they host more indigenous urban dwellers than Ward 1, which is predominantly a government reservation area for offices and other establishments. Thereafter, 11 out of the 16 clusters from the two Wards were randomly selected and all households within the selected clusters were visited to recruit eligible participants.

The study population consisted of consenting adults aged 50 years and above. Participants with arthritis, nerve injuries, previous cerebrovascular events with focal neurologic deficits, or severe chronic (end-stage) illnesses in the last three months were excluded from the study. A total of one thousand and thirty-one (1031) participants were enrolled and taken through a battery of cardiovascular, cognitive, and frailty assessment tools. Ethical approval was obtained from the University of Ibadan/University College Hospital (UI/UCH) Health Research Ethics Committee (HREC). Measures of socio-demography, physical performance, vascular risk factors, blood tests, blood pressure and clinical-related variables were obtained by research assistants and medical practitioners. Participants using tobacco and alcohol at the time of data collection and those who had quit in the last 12 months beforehand were classed together. BMI was calculated from the height and weight measured using a stadiometer and weighing scales.

Cognitive function was assessed using translated and validated neuro-psychometric instruments: Montreal Cognitive Assessment [17], and the Identification and Intervention for Dementia in Elderly Africans (IDEA) cognitive screen [18]. The IDEA and MoCA are tests of general cognitive functioning that have been well-validated in the African setting. The clinical frailty scale (CFS) score was calculated using the Rockwood scale [19]. It is a 9-point clinical scale (1 – 9) with higher scores representing worsening clinical frailty (1: very fit, 2: well, 3: managing well, 4: vulnerable, 5: mildly frail, 6: moderately frail, 7: severely frail, 8: very severely frail, and 9: terminally ill). Participants’ HGS was obtained using a dynamometer for both left and right hands. Each participant was asked to repeat the exercise twice for each hand and the better of the two measurements were used. The grip strength for each participant was defined as the highest recorded/maximum HGS (max. HGS) of both hands.

All data were entered into RED-Cap and analyzed using STATA Version 18.0. Descriptive statistics were computed for continuous and categorical variables and test of normality carried out using the Shapiro-Wilks test. The Pearson chi-square test, Fisher’s exact, and independent student *t*-test were used to analyze sex differences in variables. To determine the normative values of HGS, the study population was stratified by gender and then by age into five-year subgroups from 50 years until the age of 80 years and over. Analyses of grip strength were undertaken by age and gender and were presented by left hand, right hand and average. Mean (SD) of max. HGS in kilograms was calculated as the data for each group was normally distributed. Linear regression analysis was used to investigate the relationship between max. HGS and socio-demographic characteristics, behavioral/lifestyle factors, cardiometabolic features, cognitive performance as well as clinical frailty. The variables included in the regression model were identified following literature search [4,6,7,9,20]. Subgroup analysis was then performed. Corresponding beta coefficients and p values were reported. The level of statistical significance was set at 0.05.

## 3 Results

### 3.1 Characteristics of the study population and hand grip strength (HGS)

Of the studied 607 participants, there were 197 (32.2 %) males and 410 (67.8 %) females Baseline socio-demographic and clinical details are shown in Table S1. The age ranged from 50 – 100 years, the mean age (SD) was 64.6 (11.5) years. The mean (SD) max. HGS (in kg) of the study population was 19.65±10.16 overall; 24.51(14.01) among males, and 17.31(6.51) among females (*p* < 0.001). Overall, the difference in the max. HGS between males and females was 7.20 kg (95 % CI: 5.55 – 8.83 kg) which was statistically significant (*p* < 0.001). The normative values of HGS stratified by sex and age group are as shown in Table 1. Max. HGS decreased with age group, *F* = 10.43, *p* < 0.001 as shown in Figure S1A. The variation in max. HGS across age group did not differ significantly between males and females (*p* = 0.175). Figure S1B

**Table 1:** Normative data for Hand Grip Strength (kg): age and sex-stratified.

Men					Women				
Age	N	Right	Left	Average	Age	N	Right	Left	Average
50 – 54	37	23.1 (8.7)	22.2 (8.0)	22.7 (8.1)	**50 – 54**	72	19.6 (7.6)	18.3 (7.5)	18.9 (7.3)
55 – 59	28	25.6 (9.7)	23.7 (8.1)	24.7 (8.7)	**55 – 59**	49	17.6 (6.0)	16.7 (6.3)	17.1 (6.0)
60 – 64	31	25.1 (10.0)	22.2 (9.0)	23.7 (9.2)	**60 – 64**	49	17.3 (5.8)	15.4 (5.3)	16.4 (5.3)
65 – 69	19	22.5 (5.9)	21.9 (6.1)	22.2 (6.0)	**65 – 69**	34	17.4 (5.9)	15.8 (5.5)	16.6 (5.4)
70 – 74	14	24.5 (3.9)	23.3 (3.9)	23.9 (3.4)	**70 – 74**	48	15.5 (5.1)	14.4 (4.8)	14.9 (4.7)
75 – 79	15	19.3 (7.8)	17.3 (8.2)	18.3 (7.7)	**75 – 79**	21	15.1 (4.8)	13.8 (4.2)	14.5 (4.3)
≥ 80	15	16.0 (7.2)	15.9 (6.2)	16.2 (6.6)	**≥ 80**	51	12.1 (4.6)	10.7 (3.9)	11.4 (4.1)

### 3.2 Determinants of HGS (unadjusted and adjusted linear regression analysis)

In the unadjusted analyses (Table 2), age, sex, years of education, marital status, smoking, and alcohol use were significantly related to max. HGS. Presence of dyslipidemia (β −1.33, p-value = 0.122) and diabetes mellitus (β −1.82, p-value = 0.323) showed a trend of negative association with max. HGS, while clinical frailty (β= - 2.46), higher MoCA (β=0.61) and IDEA scores (β=0.98) showed significant relationships (Table 2). In the adjusted analyses, female sex (β=−6.38, *p* < 0.001), CFS score (β=−1.35, p 0.001), and baseline MoCA score (β=0.37, *p* < 0.001) showed significant association with max. HGS. In the subgroup analysis, in addition to the MoCA score and CFS score, presence of hypertension (β=2.64, *p* < 0.001) and diabetes mellitus (β=−2.61, p 0.05) were independently associated with max. HGS among females, but not males.

**Table 2:** Linear regression analysis showing the correlates/determinants of maximum hand grip strength (max. HGS).

	All		Male		Female	
	Unadjusted beta (p value)	Adjusted beta (p value)	Unadjusted beta (p value)	Adjusted beta (p value)	Unadjusted beta (p value)	Adjusted beta (p value)
Age (years)	−0.19(<0.01)	0.01(0.974)	−0.20(0.031)	0.11(0.428)	−0.18(<0.001)	−0.02(0.541)
Sex (female)	−7.19(<0.01)	−6.38(<0.001)*	–		–	
Years of education	0.57(<0.01)	−0.11(0.325)	0.45(0.053)	0.05(0.849)	0.33(<0.001)	−0.19(0.025)*
Marital status						
Widow/widower	−6.02(<0.01)	−1.06(0.320)	−7.26(0.019)	−5.68(0.115)	−3.36(<0.01)	−0.66(0.364)
Separated/Divorced	−2.30(0.052)	−0.52(0.686)	−0.47(0.855)	0.34(0.911)	−2.83(0.005)	−1.21(0.233)
Hypertensive (yes)	−0.21(0.805)	1.22(0.178)	−1.69(0.399)	−2.34(0.340)	1.69(0.012)	2.64(<0.001)*
BMI (kg/m^2^)	−0.06(0.283)	0.07(0.199)	0.14(0.368)	0.08(0.642)	0.18(<0.001)	0.08(0.053)
Diabetic (yes)	−1.82(0.323)	−1.26(0.508)	1.90(0.691)	2.90(0.608)	−2.84(0.041)	−2.61(0.050)*
Smoking (yes)	2.79(0.030)	−2.76(0.065)	−1.99(0.367)	−3.94(0.134)	−0.85(0.666)	−3.07(0.157)
Alcohol use	4.23(<0.001)	0.91(0.386)	0.64(0.764)	3.02(0.257)	1.53(0.056)	0.69(0.386)
Dyslipidaemia (yes)	−1.33(0.122)	−0.46(0.599)	−2.87(0.206)	−3.56(0.151)	0.51(0.436)	0.83(0.176)
MoCA score	0.61(<0.001)	0.37(<0.001)*	0.56(0.003)	0.36(0.205)	0.45(<0.001)	0.36(<0.001)*
IDEA score	0.98(<0.001)	0.13(0.477)	0.93(0.014)	−0.01(0.971)	0.76(<0.001)	0.21(0.115)
CFS score	−2.46(<0.001)	−1.35(0.009)*	−3.18(0.002)	−2.98(0.032)*	−1.64(<0.001)	−0.77(0.042)*

⁎ significant (p value <0.05). CFS, Clinical Frailty Scale; BMI, Body Mass Index; MoCA, Montreal Cognitive Assessment, IDEA, Identification and Intervention for Dementia in Elderly Africans.

## 4 Discussion

Among this cohort of older Nigerian Africans, we determined the normative values of HGS and identified its unique determinants with female sex, clinical frailty, and baseline cognitive performance observed to be independently associated with max. HGS. In the subgroup analysis, besides cognitive function and frailty, presence of hypertension and diabetes mellitus showed significant association with max. HGS among females, but not males. We observed that the mean max. HGS of 19.65 kg is lower than findings from other studies in Nigeria and sub-Saharan Africa as well as reports from the European/Caucasian-ancestry population [4,6,9]. Our findings are however consistent with data from the HAALSI cohort where mean max. HGS in the African population was noted to be lower than in the Western population [20].

The lower mean max. HGS in our study is attributable to the higher age of our study population, a predominantly female population, ethno-racial characteristics as well as the near-normal mean BMI (26.49±7.27 kg/m2) which may be reflective of poor nutritional status, a key contributor to muscle mass [21]. In our study, the independent relationship between clinical frailty, cognitive function, and HGS further buttresses the intimate link between frailty, physical health, and brain health. Emerging evidence posits that both cognitive and motor function share similar neuro-cortical pathways such that, any disruption in the shared neural axis may result in concomitant disruption of optimal cognitive and motor performance [22]. In addition, white matter hyperintensities, a known pathological substrate of neurocognitive dysfunction, have been associated with sarcopenia [23].

Among female participants, besides cognitive status and clinical frailty, we found a significant association between max. HGS, and presence of hypertension and diabetes mellitus having adjusted for other variables. This finding was not observed among male participants. This is consistent with reports from other studies [[24], [25], [26]] and a recent population-based study [27]. Of note, high blood pressure has been linked with higher HGS especially amongst older adults and among persons with higher adiposity [25,26]. Some authors have suggested that the relationship between high HGS and hypertension may be mediated by BMI [28,29]. Indeed, in our study, participants with hypertension had a significantly higher mean (SD) BMI 27.10±7.40 compared to normotensives 25.44±6.90. In addition, overall, we observed a trend of an inverse relationship between dyslipidemia, diabetes mellitus and HGS, further underlying the link between HGS and other traditional vascular risk factors [24].

While HGS showed a positive association with BMI among females in this study, we did not observe a significant association among males. Low HGS has been similarly linked with low BMI in studies carried out in rural Ghana and South Africa [7,8]. The BMI is seen as a measure of nutritional status, and this may explain the relationship with frailty [21]. In a recent cross-sectional study of adults older than 60 years at a geriatric center in Ibadan, Nigeria, obesity was found to have an independent, protective association with frailty [30]. This is similar to findings in a prospective cohort study where being underweight was found to be significantly associated with frailty [31].

Our study has several strengths. The use of a population-based sample implies that the results are likely to be generalizable to a range of older Nigerians living in urban slums. However, there is a need to determine if the pattern will be similar to values in urban areas due to the difference in health-related behaviors. In this study, HGS was measured twice, given the possibility of physiological variability. The better of the two measurements was used and the maximum HGS of both hands documented. Besides, participants recruited in this study were in a fairly good state of health, and this is expected to reduce variability, giving a better characterization of the HGS. Some weaknesses should however be highlighted. Being a review of baseline data, causality between HGS, cognitive as well as vascular risk markers could be not inferred. Nutritional status was assumed using the participants' BMI. A more affirmative message regarding handgrip and nutritional status could require documentation of dietary composition over time.

## 5 Conclusion and future directions

HGS, an inexpensive, non-invasive indicator of the risk of neurocognitive decline as well as a surrogate marker of cardiovascular disease incidence and mortality, is lower among this population of older Nigerians. The variations in HGS may be explained by specific population demographics, prevalence of multi-morbidities, geographic setting, and nutritional factors as well as ethno-racial and genotypic variations. In this cohort, female sex, clinical frailty, and baseline cognitive performance were independently associated with HGS overall. In addition to clinical frailty and cognitive status, presence of hypertension and diabetes mellitus showed significant association among female participants. Understanding population-specific determinants of HGS is necessary for the development of low-cost preventive or therapeutic interventions that may help improve overall physical, cardiometabolic, and cognitive health. Future longitudinal studies should espouse the link between HGS, nutritional status, cardiometabolic, and neuroimaging biomarkers with cognitive status.

## Declaration of generative AI and AI-assisted technologies in the writing process

The authors have not used AI or any AI-assisted technologies in the writing process

## CRediT authorship contribution statement

**Rufus O. Akinyemi:** Writing – review & editing, Writing – original draft, Visualization, Validation, Supervision, Software, Resources, Project administration, Methodology, Investigation, Funding acquisition, Formal analysis, Data curation, Conceptualization. **Oladotun V. Olalusi:** Writing – review & editing, Writing – original draft, Visualization, Validation, Supervision, Software, Project administration, Methodology, Investigation, Formal analysis, Data curation. **Gabriel O. Ogunde:** Writing – review & editing, Visualization, Validation, Supervision, Project administration, Investigation, Formal analysis, Data curation. **Tolulope O. Akinyemi:** Writing – review & editing, Visualization, Validation, Software, Resources, Project administration, Data curation. **Joseph O. Yaria:** Writing – review & editing, Writing – original draft, Visualization, Validation, Supervision, Software, Resources, Project administration, Methodology, Investigation, Funding acquisition, Formal analysis, Data curation, Conceptualization. **Olabode Oguntiloye:** Writing – review & editing, Visualization, Supervision, Software, Resources, Formal analysis, Data curation. **Ayotomiwa Fagbemi:** Writing – review & editing, Validation, Supervision, Software, Resources, Data curation. **Eniola O. Cadmus:** Writing – review & editing, Validation, Software, Resources, Project administration, Funding acquisition, Formal analysis, Data curation. **Femi O. Popoola:** Writing – review & editing, Resources, Funding acquisition, Formal analysis, Data curation, Conceptualization. **Mayowa Ogunronbi:** Writing – review & editing, Validation, Supervision, Software, Resources, Project administration. **Dorcas Olujobi:** Writing – review & editing, Visualization, Validation, Supervision, Software, Project administration. **Olaoluwa Famuyiwa:** Writing – review & editing, Software, Resources, Project administration, Methodology. **Joshua O. Akinyemi:** Writing – review & editing, Software, Resources, Project administration, Methodology, Investigation, Funding acquisition, Formal analysis, Data curation, Conceptualization. **Mayowa O. Owolabi:** Writing – review & editing, Writing – original draft, Visualization, Validation, Supervision, Project administration, Investigation, Funding acquisition, Formal analysis, Data curation, Conceptualization. **Roman Romero-Ortuno:** Writing – review & editing, Visualization, Validation, Supervision, Software, Resources, Project administration, Methodology, Investigation, Funding acquisition, Formal analysis, Data curation, Conceptualization. **Adesola Ogunniyi:** Writing – review & editing, Writing – original draft, Visualization, Validation, Supervision, Software, Resources, Project administration, Methodology, Investigation, Funding acquisition, Formal analysis, Data curation, Conceptualization. **Raj Kalaria:** Writing – review & editing, Writing – original draft, Visualization, Validation, Supervision, Software, Resources, Project administration, Methodology, Investigation, Funding acquisition, Formal analysis, Data curation, Conceptualization. **Brian Lawlor:** Writing – review & editing, Writing – original draft, Visualization, Validation, Supervision, Software, Resources, Project administration, Methodology, Investigation, Funding acquisition, Formal analysis, Data curation, Conceptualization.

## Declaration of competing interest

The authors have nothing to disclose and there are no conflicts of interests

## Appendix Supplementary materials

Image, application 1
