# Supplementary material for: Correlates of hand grip strength in a cohort of older Nigerian Africans: Findings from the population-based VALIANT project
Source: J Frailty Aging. 2025 Sep 3;14(5):100068. doi: 10.1016/j.tjfa.2025.100068 (PMC12444469; doi:10.1016/j.tjfa.2025.100068)
Supplement: Supplementary file 1 [file mmc1.docx]

# **Correlates of Hand grip strength in a cohort of older Nigerian Africans: Findings from the population-based VALIANT Project**

AUTHORS AND AFFILIATIONS

Rufus O. Akinyemi, MBBS, PhD1,2,3*, Oladotun V Olalusi, MD 1,2, Gabriel O. Ogunde, MSc3, Tolulope O Akinyemi, MSc, FMLSCN4, Joseph O Yaria, MBBS MSc2, Olabode Oguntiloye MBBS2, Ayotomiwa Fagbemi MBBS 2, Eniola O Cadmus, MBBS, PhD3, Femi O. Popoola, MBBS, PhD5, Mayowa Ogunronbi MPH1, Dorcas Olujobi MSc1, Olaoluwa Famuyiwa BSc1, Joshua O. Akinyemi, PhD5, Mayowa O. Owolabi, DSc2,3, Roman Romero-Ortuno, MD, PhD6, Adesola Ogunniyi, MD, FAS2,3,7 Raj Kalaria, DSc 8, Brian Lawlor, MD 9,10,11

(1) Neuroscience and Aging Research Unit, Institute of Advanced Medical Research and Training, College of Medicine, University of Ibadan, Ibadan, Nigeria,

(2) Department of Neurology, University College Hospital, Ibadan, Oyo, Nigeria,

(3) College of Medicine, University of Ibadan, Ibadan, Oyo, Nigeria

(4) Lead City University, Ibadan, Oyo, Nigeria,

(5) Department of Epidemiology and Medical Statistics, College of Medicine, University of Ibadan, Ibadan, Nigeria,

(6) Global Brain Health Institute, Trinity College, Dublin, Ireland,

(7) African Dementia Consortium, Ibadan, Ibadan, Nigeria,

8) Translational and Clinical Research Institute, Newcastle University, Campus for Ageing & Vitality, Newcastle Upon Tyne NE4 5PL, United Kingdom.

(9) Global Brain Health Institute, Trinity College Dublin, Dublin, Ireland,

(10) Trinity College Institute of Neuroscience, School of Psychology, Trinity College Dublin, Dublin, Ireland

(11) St James Hospital, Dublin, Ireland

*Corresponding author:

Prof. Rufus Olusola Akinyemi MBBS (Ib), MSc (Ib), PhD (Newcastle), MWACP, FMCP (Neurol), FGBHI, FAMedS, FAS.

Professor and Consultant Neurologist, Neuroscience and Ageing Research Unit, Institute for Advanced Medical Research and Training, College of Medicine, University of Ibadan, University College Hospital Campus, Queen Elizabeth Road, Ibadan, Nigeria.

E mail: rufusakinyemi@yahoo.com; roakinyemi@com.ui.edu.ng; rufusakinyemi@gbhi.org

Mobile: +234 (0) 8033 704 384

**KEYWORDS:** Hand grip strength (HGS), Frailty, Nigerian-Africans, Indigenous Africans, Cognitive health, Physical health, Physical fitness, LMICs; muscular strength

**WORD COUNT:** Abstract (206), manuscript (2028) Tables 1 and 2; Supplementary tables S1 and S2, Figures S1A/B

Table S1: Socio-demographic Variables of Study Population (N=607)

|  | Male (n=197; 32.5%) | Female (n=410; 67.5%) | Total (N= 607) | p-value |
| --- | --- | --- | --- | --- |
| Age (Mean$\boldsymbol{\pm}$SD) | 64.4$\pm$10.7 | 64.7$\pm$11.9 | 64.6$\pm$11.5 | 0.807 |
| Years of education | 8.2$\pm$4.4 | 4.6$\pm$4.6 | 5.6$\pm$4.8 | <0.001 |
| Religion |  |  |  |  |
| Christianity | 82(41.8) | 197(48.2) | 279 | 0.144 |
| Islam | 114(58.2) | 212(51.8) | 326 |  |
| Ethnicity |  |  |  |  |
| Yoruba | 192(97.5) | 402(98.0) | 594 | 0.640 |
| Non-Yoruba | 5(2.5) | 8(2.0) | 13 |  |
| Marital status |  |  |  |  |
| Currently married | 137(69.5) | 153(37.3) | 290 | <0.001 |
| Widow/widower | 24(12.2) | 204(49.8) | 228 |  |
| Others | 36(18.3) | 53(12.9) | 89 |  |
| Hypertensive (yes) | 109(55.3) | 271(66.1) | 380 | 0.010 |
| BMI (kg/m^2^) | 24.15$\pm$6.31 | 27.61$\pm$7.43 | 26.49$\pm$7.27 | <0.001 |
| Diabetic (yes) | 9(4.6) | 23(5.6) | 32 | 0.591 |
| Smoking (yes) | 59(30.7) | 11(2.7) | 70 | <0.001 |
| Max. HGS (Mean$\boldsymbol{\pm}$SD), kg | 24.51(14.01) | 17.31(6.51) | 19.65(10.16) | <0.001 |
| Alcohol use (yes) | 135(68.5) | 82(20.2) | 217 | <0.001 |
| Dyslipidaemia (yes) | 52(26.4) | 162(39.5) | 214 | 0.002 |
| MoCA score | 21.30$\pm$5.72 | 16.91$\pm$6.41 | 18.10$\pm$6.53 | <0.001 |
| IDEA score | 11.82$\pm$3.21 | 10.87$\pm$3.28 | 11.13$\pm$3.29 | <0.001 |
| CFS score | 2.52$\pm$1.04 | 2.71$\pm$1.01 | 2.65$\pm$1.01 | 0.042 |
| CFS score median (Q1, Q3) | 2(2, 3) | 3(2, 3) | 3(2, 3) | 0.004^a^ |

BMI, Body Mass Index, Clinical Frailty Scale (CFS), MoCA, Montreal Cognitive Assessment, IDEA, Identification and Intervention for Dementia in Elderly Africans ^a^, rank sum

Figure S1(A): Estimated Means of Hand Grip Strength per Age Group; F=10.43, p<0.001. Figure S1 (B): Proportion of participants with weak HGS* per Age Group; F=1.50, p=0.175 *Weak HGS was defined using a cut-off value of 18kg for males and 12kg for females (based on less than one SD of reference mean by Michael et al^1^)

REFERENCE

1. Michael AI, Ademola SA, Olawoye OA, Iyun AO, Nnabuko RE, Oluwatosin OM. Normal values for hand grip strength in healthy Nigerian adults. Nigerian Journal of Plastic Surgery. 2013 Jun 20;9(1):1–8.
